# Experimental Testing and PSO-Enhanced Neural Networks for Impact Failure Analysis of H-Section Steel Members

**DOI:** 10.3390/ma18132968

**Published:** 2025-06-23

**Authors:** Pengcheng Chen, Shuwen Bu, Lin Wang, Guoyun Lu, Jinfeng Jiao, Huiwei Yang

**Affiliations:** 1 College of Civil Engineering, Taiyuan University of Technology, Taiyuan 030024, China; chenpengcheng@tyut.edu.cn (P.C.); 20221058@llu.edu.cn (J.J.); yanghuiwei@tyut.edu.cn (H.Y.); 2 Arup International Consultants (Shanghai) Co., Ltd., Shanghai 200235, China; shu-wen.bu@arup.com; 3 Shanxi Second Construction Group Co., Ltd., Taiyuan 030024, China; tyutcost2019@126.com; 4 Department of Architecture and Civil Engineering, Lyuliang University, Lüliang 033001, China

**Keywords:** particle swarm optimization algorithm, impact experiments, machine learning, H-section steel members, multilayer perceptron

## Abstract

H-section steel members, as a commonly used load-bearing receiving member in building structures, may be subjected to the impact of accidental loads during their service life, and therefore, the impact loads need to be considered when carrying out the design. In this paper, based on experimental testing, the particle swarm optimization algorithm (PSO) is used to optimize the hyperparameters of the multilayer perceptron (MLP), and a combined prediction model PSO-MLP for H-section steel members subjected to lateral impact loads is proposed to predict the damage of the H-section steel members after impact. The results show that the prediction model based on PSO-MLP can predict the damage of the H-beam columns more accurately, and compared to the random forest model (RF) and the support vector machine (SVM), the PSO-MLP model has better prediction accuracy and robustness. In addition, the effects of different features on the impact performance of the members were analyzed, in which the weakest impact location is 0.57 L away from the fixed end and the effects of axial compression ratio, flange, and web thickness were similar to the results of previous studies; the impact angle showed a strong nonlinear relationship with the critical impact velocity, which the weakest impact angle is around 50° from the strong axle; and the height and width of the cross-section showed a linear enhancement of the impact performance.

## 1. Introduction

With the rapid development of modern architecture and engineering structures, H-section steel members are widely used in various structural systems due to their superior mechanical properties and economic benefits [1,2]. However, during the service life of a building structure, it may be hit by vehicles or other accidental loads [3,4,5,6,7], which seriously affect the safety of the structure and endanger life and property. Therefore, the impact of these extreme loads needs to be considered in structural design. Under the impact load, the structure undergoes nonlinear large deformation and continuously accumulates damage, which seriously affects the subsequent bearing capacity of the structure. Therefore, it is particularly important to study the dynamic performance of structural members under impact loads and further rationalize the design of the structure to improve its ability to resist continuous collapse.

Zhao [8] and Wang [9] conducted impact tests and axial compression tests on 13 H-section steel members and found that the local deformation generated in the impact zone of H-section steel members has a significant impact on the bearing capacity of steel columns, and axial load significantly reduces the impact resistance of steel columns. They proposed an empirical formula to evaluate the remaining bearing capacity of the H-section steel members after impact. Huo et al. [10] studied the dynamic response and catenary effect of welded H-shaped steel beams under impact load through a combination of experimental and numerical research methods and focused on the influence of impact energy and flange width to thickness ratio on the dynamic response of steel beams. Xiang [11] and Zhou [12] studied the dynamic response of H-shaped steel in steel garages under vehicle impact, including the effects of collision speed, load-bearing capacity, vehicle type, etc., on the structural impact resistance performance. They also introduced the vehicle energy absorption ratio and improved the impact resistance design formula of members in European standards. Makarem [13] studied the nonlinear behavior of high-strength H-shaped steel under strong axial impact, analyzed the impact resistance of steel columns under different impact positions, impact velocities, impact block masses, and axial compression ratios, and found that overall plastic failure was the main failure type, and the deformation of the column flange occurred before the overall plastic failure. Wei-hui et al. [14] investigated the influence of different scale ratios on the dynamic response of H-section beam-column substructures subject to impact loads with numerical simulations. The changes in the mass and velocity of the drop hammer on the impact force, impact time history, vertical displacement, internal force, and energy absorption of the structure were studied.

Although numerous scholars have conducted extensive research on the impact resistance performance of H-shaped steel members, the main focus was on experimental research and numerical simulation [15,16]. While the traditional experimental methods can provide intuitive information about member performance, due to limitations in experimental conditions, it is difficult to fully reveal all the potential influencing factors. Some empirical formulas for evaluating the impact resistance of H-shaped steel can be obtained through experimental research [17], but the current empirical formulas cannot include all the factors that need to be considered in the design. In recent years, with the rapid progress of computing technology, deep learning has been widely applied in many fields due to its powerful nonlinear capture ability and large-scale dataset processing ability, such as structural health monitoring [18,19,20,21,22], material performance prediction [23,24,25,26,27], structural optimization design [28,29,30,31,32], and so on. In these research processes, researchers often use a large amount of experimental data and neural network algorithms to train corresponding models. For the neural network models, determining the optimal set of hyperparameters to enhance the accuracy of the prediction model and prevent overfitting is a time-consuming and laborious task. Therefore, researchers usually use a combination of optimization algorithms and deep learning models to solve this problem [33,34,35,36,37]. In addition, due to the inability of machine learning models to directly output patterns and explanations in training data, researchers use model interpretation algorithms, feature importance, or partial dependency graphs to improve the interpretability of machine models.

**Figure 1:**
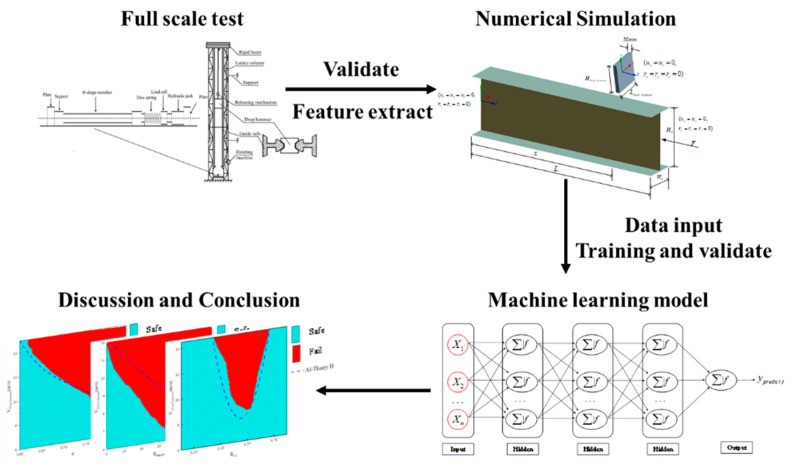
Workflow of study.

This study performs a combined research method of experimental research, numerical simulation, and machine learning to investigate the impact resistance performance of H-section steel members. Section 2 introduces the methodology of the test, numerical simulation, and PSO-MLP model. Section 3 introduces the results of the test and simulation, and the frame of the PSO-MLP model that was used in the Section 4. Section 4 discusses the influence of features on the failure of the member. Section 5 concludes the innovation of this study and the key findings. The overall process map is shown in Figure 1. The purpose of this study is to provide engineering and technical personnel with research methodologies to better understand the factors influencing the impact resistance of H-shaped steel members, thereby offering strong support for their design and application.

## 2. Experimental and Numerical Methods

From the above research, it can be seen that the application status and potential of machine learning (ML) technology in civil engineering are covered by research and application in many aspects, such as structural damage prediction, structural design parameter optimization, and evaluation of structures under specific loads. However, in the research field of impact resistance of steel members, the application of machine learning (ML) technology is still in the preliminary stage. Moreover, currently, the ML technology is mostly used for structural parameter optimization in anti-impact design, which has no essential difference from conventional topology optimization, while there is a relative lack of analysis on the use of numerical model results, as the research method of the ML model dataset. Therefore, the impact resistance performance of H-section steel members was explored using a combined research method of experimental research, numerical simulation, and machine learning in this study. The experiment of the H-shaped members subjected to impact loading is introduced in this section, which is used to verify the effectiveness of the numerical model.

### 2.1. Impact Test

**Figure 2:**
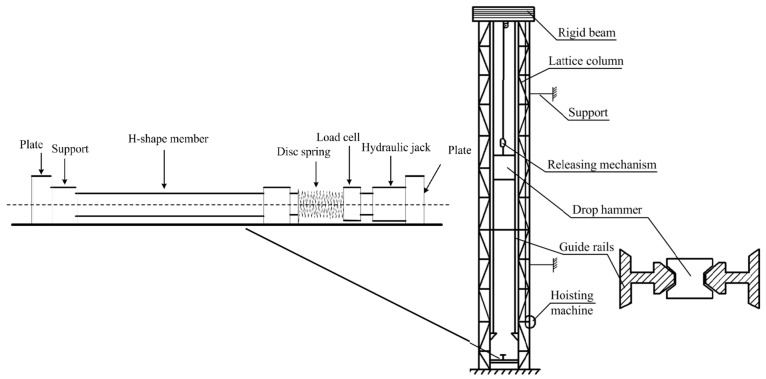
Schematic diagram of the experimental setup.

The experiment, conducted by the authors of [38], was performed on the DHR9401 large drop hammer impact testing machine independently developed and designed by Taiyuan University of Technology, as shown in Figure 2. The impact mass of the experiment is 251.02 kg, and the impact position is 1/3 of the distance between the member and the constraint end. An impact force sensor was placed between the impact head and the weight to record the time history curve of the impact force. As shown in Figure 2, the axial force of the member is applied by a displacement-controlled 50 t jack, and the axial force can be continuously loaded on the member during the impact process through a disk spring to prevent the axial force from disappearing instantly during the impact process. To better ensure the boundary conditions of one end fixed and one end sliding of the member, the fixed-end support uses high-strength bolts to connect the welded pad at the end of the member to the experimental platform.

**Figure 3:**
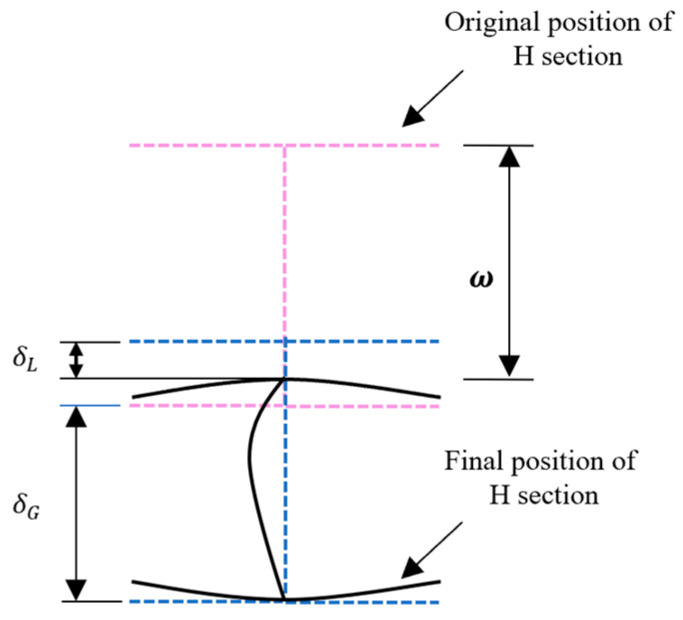
Schematic diagram of member section deformation.

The members of this experiment were welded with a Q355B steel plate, and the mechanical properties of the steel are shown in Table 1. The standard tensile test was conducted in accordance with GB/T228-2010 [39]. The cross-sectional parameters, length, and drop hammer parameters of the members are shown in Table 2. The impact experiment was divided into two stages: in the first stage, an axial load N is applied to the member, which remains constant after reaching the predetermined axial compression ratio; then, the impact load is applied in the second stage. After the experiment, the local and overall deformation results of the members were measured, as shown in Figure 3. The results of member deformation and impact force were taken as the validity of the numerical simulation results of this study, which ensured the scientific validity of the research methods and results.

### 2.2. Data Source and Feature Projection

The dataset required for this study was obtained through the batch numerical simulation. By simulating different impact conditions and cross-sectional geometric parameters of H-beams, a large number of samples were obtained for training the machine learning models and verifying their accuracy. Due to the complex impact conditions of H-shaped steel, to simplify it appropriately, this study assumed that the boundary conditions of the member were fixed at one end and sliding at the other end, with a length of 3 m; the steel strength grade of the member remains unchanged, and the drop hammer was a hexahedron. The following 10 widely studied features were selected in this study, including 6 external load features (hammer height-*H_drop hammer_*, hammer length-*L_drop hammer_*, ratio of the distance from the impact position to the fixed end to the total length-*R_x/L_*, impact speed-*V_drop hammer_*, impact angle-*R_impact_*, and axial compression ratio-*n*) and 4 cross-sectional features (H-section width-*W_H_*, H-section height-*H_H_*, flange thickness-*T_flange_*, and web thickness-*T_web_*). The range of values for each feature is shown in Table 3. These characteristic values are taken and matched to generate 7000 impact conditions. The established numerical model is used for calculation, and the simulation results are divided into two types: member failure and non-failure.

### 2.3. Numerical Simulation

#### 2.3.1. Material Model and Basic Parameters

**Figure 4:**
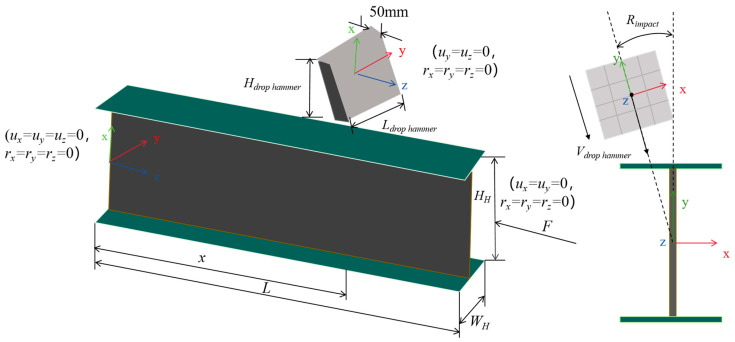
Schematic diagram of the numerical model.

A numerical model was established using Ls dyna R12 software to simulate and parameterize the experimental process, as shown in Figure 4. The H-shaped steel members are simulated using shell elements; solid elements are used to simulate the hammer, and MAT-20 material is used to set the hammer as a rigid body. The steel is described using a bilinear elastic-plastic model using MAT-3 material, where *σ*_y_ = 345 MPa, *σ*_u_ = 520 MPa, *E* = 200 GPa, *E*_h_ = *E*/100, Poisson ratio is 0.3. Using the Cowper Symonds model to consider the strain rate effect of steel, *C* and *p* are taken as 6844 s^−1^ and 3.91 [40], respectively. The drop hammer mass *m* is 251.02 kg, and the total length of the member *L* is 1500 mm. One side is set as a fixed constraint, and the other side only releases longitudinal degrees of freedom to apply axial force; The drop hammer applies an impact velocity pointing towards the centroid of the member section and defines the minimum angle between the direction of the impact velocity and the weak axis of the H-shaped steel section as the impact angle. The contact between the hammer and the steel member is set as a frictionless surface contact. When conducting batch calculation and analysis, to facilitate the calculation of the drop hammer mass, the drop hammer density is set to ρ_drop hammer_ = 3 × 10^5^ kg/m^3^.

(1) $$\sigma_{d}=\sigma_{0}[1+{\left({\dot{\varepsilon }}_{p}/C\right)}^{1/p}]$$

#### 2.3.2. Grid Sensitivity

**Figure 5:**
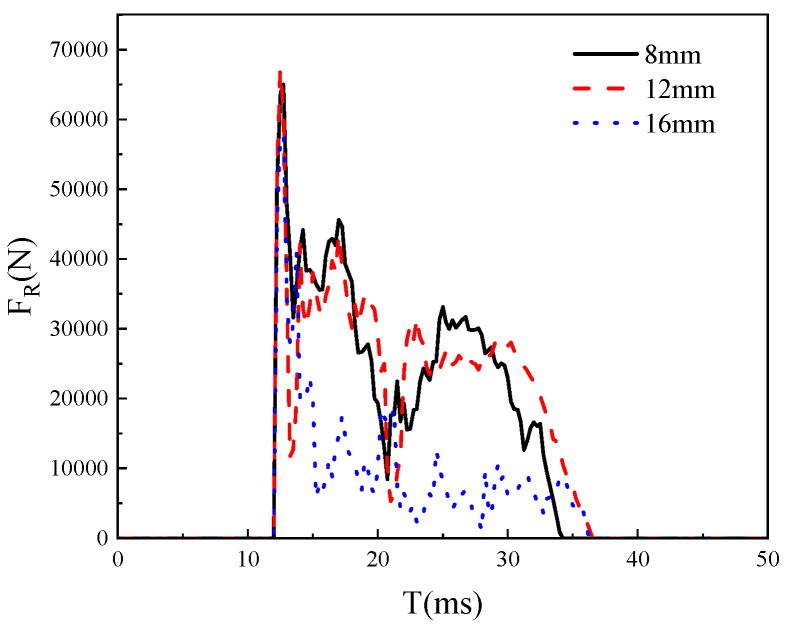
Impact force time history curves of models with different grid sizes.

**Figure 6:**
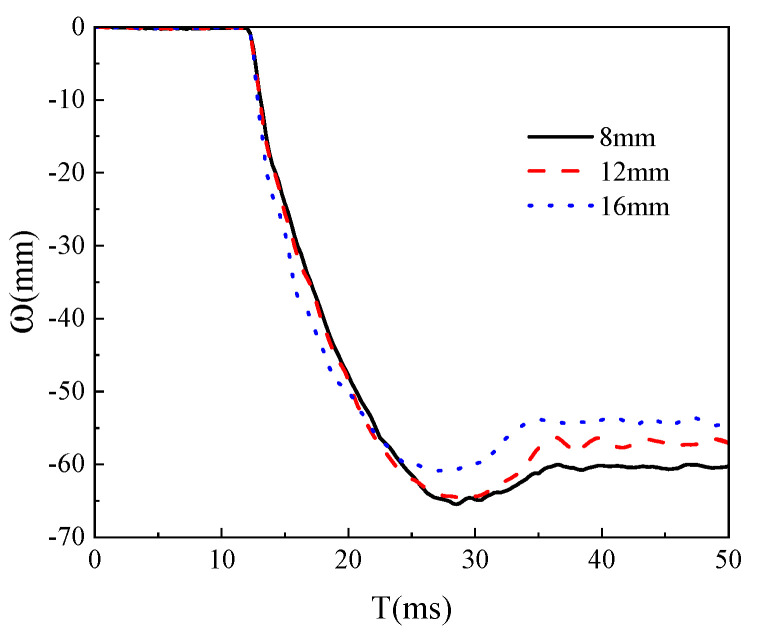
Deflection time history curves of models with different grid sizes.

Choosing the appropriate grid density is one of the important factors to consider in this study. A good grid can provide the most accurate results with a reasonable calculation time. Using a particularly fine grid can significantly increase calculation time, but may not improve the accuracy of the results. Using a coarser grid can reduce calculation time, but lower the accuracy of the results, and the credibility of the calculation results is not high. In this study, grid models with sizes of 8 mm, 12 mm, and 16 mm were used for calculations, as shown in Figure 5 and Figure 6. The mid-span displacement curve and impact force time history curve of the models are shown in the following figure. It can be seen that there is a small difference between the calculation results of an 8 mm grid and a 12 mm grid, so the global grid size is selected as 12 mm.

### 2.4. Multilayer Perceptrons (MLP)

**Figure 7:**
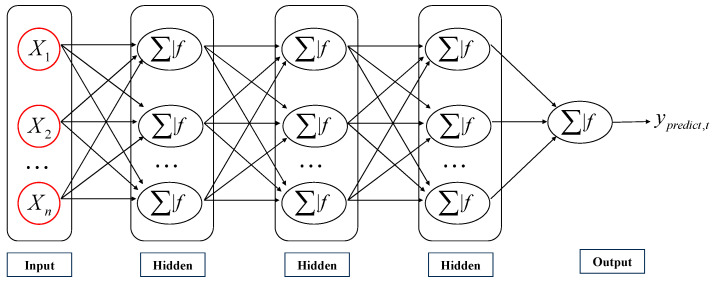
The MLP model’s basic schematics.

The MLP is a feedforward neural network and one of the most commonly used artificial neural network algorithms. As shown in Figure 7, a complete MLP algorithm consists of at least three layers: an input layer, multiple hidden layers, and an output layer, each consisting of several neurons. The function of the input layer is to extract features from the input data, with each input node corresponding to a feature. The hidden layer performs a nonlinear transformation on the input data, and its processing can be expressed as

(2) $$z_{i}=f[W_{i}⊗o_{i-1}+b_{i}]$$

where $W_{i}$ represents the weight matrix of the *i*-th layer, $o_{i-1}$ represents the output of the previous layer, $b_{i}$ represents the bias matrix of the *i*-th layer, and ⊗ represents the convolution operation between the input result and the weight matrix, adding it to the bias matrix, and then obtaining the output features of this layer through the activation function f(x).

The output layer is responsible for converting the output features of the hidden layer into the final predicted value. In this study, the *Softmax* function is used to convert the logits output by the hidden layer into a normalized probability distribution.

(3) $$O_{\mathrm{output}}=Soft\max[W_{\mathrm{output}}⊗o_{\mathrm{hidden}}+b_{\mathrm{output}}]$$

(4) $$Soft\max{\left(x\right)}_{i}=\frac{e}{\sum_{j=1}^{K}e^{Zj}}$$

where *K* represents the total number of output categories, and *Z* represents the original score of the output layer. Therefore, the MLP model can handle complex nonlinear problems, but it requires careful selection of relevant parameters to avoid overfitting or spending a lot of time on the model.

Early stopping is implemented to prevent overfitting by monitoring the validation loss during training. The key steps are as follows:

(5) $$L_{\mathrm{best}}=\underset{i\leq t}{\min}L_{\mathrm{val}}\left(i\right)$$

where *L*_val_(*t*) denotes the validation loss at epoch *t*; *L*_best_ is the minimum validation loss observed so far.

If the validation loss does not decrease for *N*_patience_ consecutive epochs, training is stopped early. The stopping condition can be expressed as

(6) $$L_{\mathrm{val}}\left(t\right)\geq L_{\mathrm{best}},\forall t\in \left[t_{0},t_{0}+N_{\mathrm{patience}}\right]$$

where *t*_0_ is the epoch when *L*_best_ was last updated.

### 2.5. Particle Swarm Optimization Algorithm (PSO)

The Particle Swarm Optimization Algorithm (PSO) is a metaheuristic algorithm, whose core idea is to find the optimal solution by simulating the foraging behavior of bird flocks. During this process, each particle will determine its next speed and position based on individual and global optima. The iterative process of particle velocity and position is as follows:

(7) $$v_{i}(t+1)=w\cdot v_{i}(t)+c_{1}r_{1}(pbest_{i}-x_{i}(t))+c_{2}r_{2}(gbest-x_{i}(t))$$

(8) $$x_{i}(t+1)=x_{i}(t)+v_{i}(t+1)$$

In the formula, *t* represents the current number of iterations; *w* is the inertia weight; *c*_1_, *c*_2_ is called the individual learning factor and social learning factor; *r*_1_, *r*_2_ is the internal random number within the range of 0 to 1, *pbest*_i_ and *gbest* are the individual best positions of particles and the global best positions of the population, respectively. When the preset number of iterations is reached, the iteration is stopped and the current optimal hyperparameters, evaluation results, and training and export of the final model are outputted.

### 2.6. Construction of MLP-PSO Coupling Model

**Figure 8:**
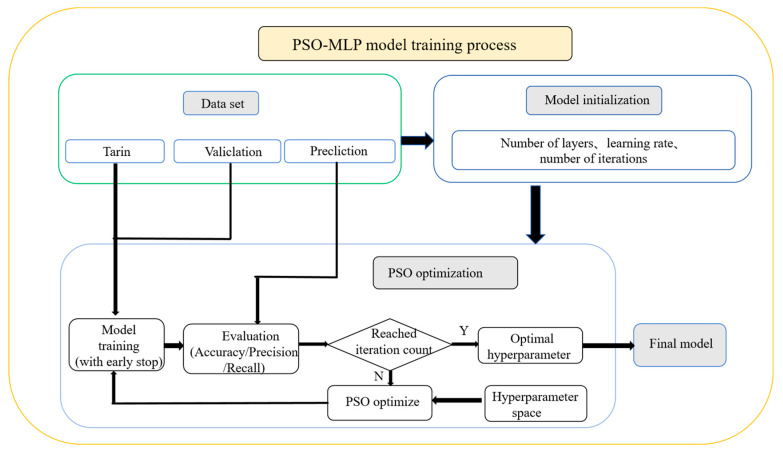
The MLP model training process framework diagram.

The training process of the MLP-PSO model is shown in Figure 8. The dataset is preprocessed and standardized for model training, with the aim of ensuring order-of-magnitude consistency of data features, thereby improving algorithm performance and reducing the impact of numerical instability. Then, the dataset is randomly divided into training sets, validation sets, and prediction sets to ensure the generalization performance of the model.

The hyperparameter space is defined based on the possible value range of the number of layers and neurons in the MLP model, which is the numerical range that PSO particles may iterate through to obtain. Simultaneously, the loss values of the training set and the verification set are used to determine the performance trend of the model during training.

(9) $$L(y,\hat{y})=-\frac{1}{n}\sum_{i=1}^{N}\left[y_{i}\log({\hat{y}}_{i})+(1-y_{i})\log(1-{\hat{y}}_{i})\right]$$

where *N* is the number of samples in the validation set; $y_{i}$ is the true label value of the i-th sample, and ${\hat{y}}_{i}$ is the model prediction value of the i-th sample.

To evaluate the accuracy and precision of the model, Accuracy, Precision, and Recall are selected as evaluation indicators, and their calculation method is as follows:

(10) $$Accuracy=\frac{TP+TN}{TP+TN+FP+FN},\;[0,\;1]$$

(11) $$Precision=\frac{TP}{TP+FP},\;[0,\;1]$$

(12) $$Recall=\frac{TP}{TP+FN},\;[0,\;1]$$

where *TP* represents the actual positive and predicted positive; *TN* represents the actual negative and predicted negative; *FP* represents the actual negative and predicted positive; *FN* represents the actual positive and predicted negative.

Accuracy is used to represent the ratio of correctly predicted sample size to total sample size, but it can be misleading when the sample results are imbalanced. Precision focuses on the accuracy of predicting positive samples, with higher values indicating lower false positives in the model. Recall represents the proportion of correctly predicted samples that are actually positive, and a higher value indicates lower false negatives.

During the model training, the batch size, that refers to the number of data samples input at once during model training is also an important hyperparameter. The batch size will directly affect computational efficiency, memory usage, and model optimization effectiveness. A larger batch size is more accurate in calculation, but it requires more memory and may lead to a decrease in generalization ability. A batch size of 72 was selected, taking into account the number of samples in the training set and the results of pre-training.

**Figure 9:**
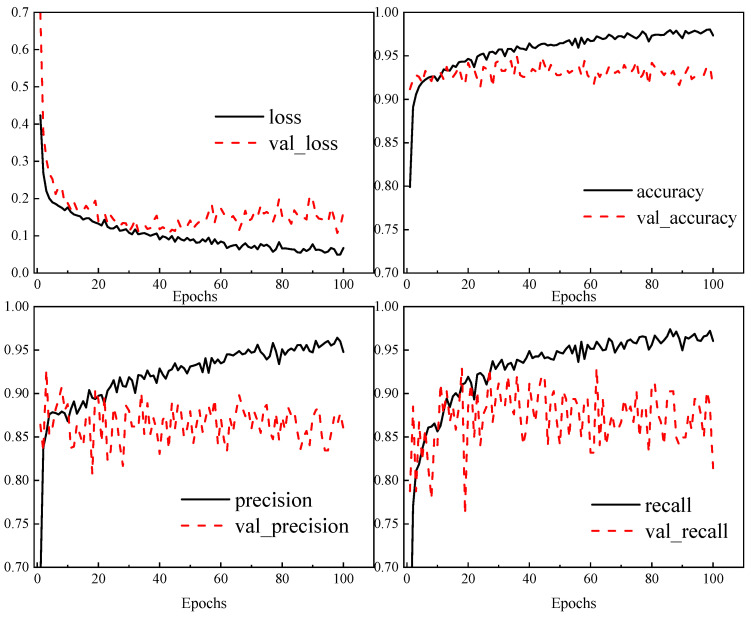
Model training metric graph.

However, during the training process, it was found that the model had overfitting, as shown in Figure 9. When the training cycle reaches 50 times, the validation loss of the model significantly deviates from the training loss, and the accuracy and precision of the model also show a certain decrease, indicating that the model has serious overfitting. Therefore, an early stop strategy is added during each training process of the model to prevent overfitting. When the loss does not decrease in multiple training cycles, the current training is ended, and the accuracy and accuracy of the current training model are evaluated to determine whether the current hyperparameter is better than the previous one. After several iterations, if the hyperparameter is not changed, which means the optimal parameters are obtained, the final MLP model is trained and saved, and then the MLP model is called to predict the new data.

## 3. Results

### 3.1. Experimental and Numerical Simulation Results

In this study, the 11 H-shaped steel members’ impact tests were conducted, and the local and overall deformation results of the members are shown in Table 4. At the same time, corresponding numerical simulation results and simulation errors were provided. It can be seen that the error between numerical simulation and experimental results is within 10%, indicating that the numerical model can better reflect the deformation response of members under impact loads.

**Figure 10:**
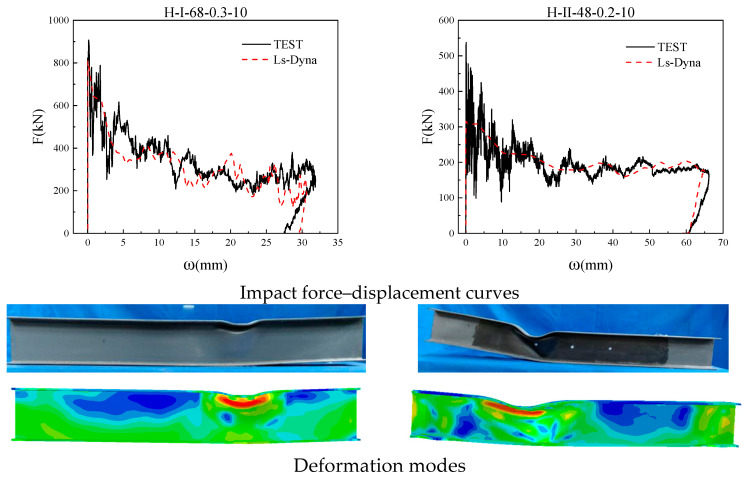
Comparison chart with experimental members.

**Figure 11:**
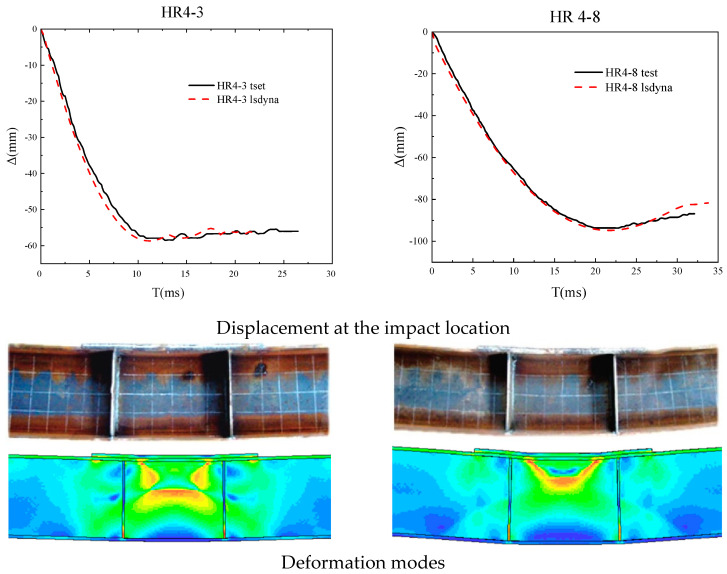
Comparison chart with Huo et al. experimental members [41].

To ensure the accuracy of the numerical model, two members from this experiment and two members from Huo et al. [41] were selected for comparison of displacement time history curves. It can be seen from Figure 10 and Figure 11 that the simulation results are in good agreement with the experimental results in terms of the deformation morphology and force–displacement curve of the members. It can be explained that the numerical model established in this article is relatively reasonable and can accurately simulate the deformation process of H-shaped steel members under impact.

### 3.2. Summary of Numerical Simulation Calculation Results

**Figure 12:**
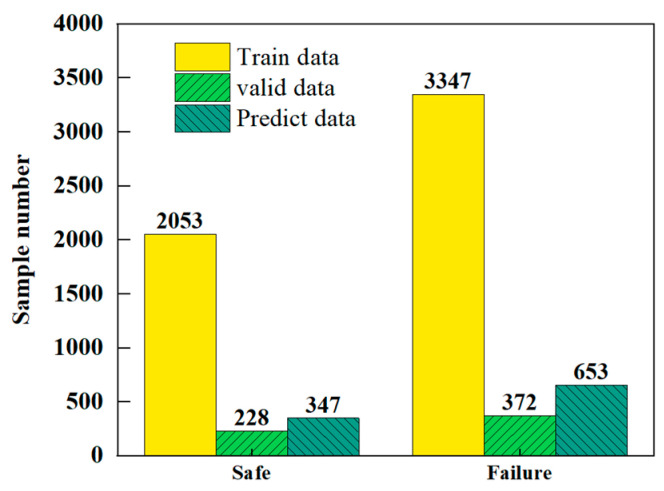
Distribution plot of sample results.

The numerical simulation batch calculation results were summarized and sorted to obtain the dataset distribution diagram, as shown in Figure 12. Among the 7000 samples, the proportion of failed members was 28.5%, and the proportion of non-failed members was 71.5%. Although the sample data exhibited a certain degree of imbalance, appropriate performance metrics were used during the machine learning model training, and the MLP algorithm performed better on imbalanced data.

At the same time, to further demonstrate the predictive accuracy of the MLP model, PR, RF, SVM, and KNN algorithms were used to establish corresponding machine learning models based on the same dataset, and the prediction set was used as the computational dataset for the model performance indicators. The performance comparison of different models is shown in Table 5. Compared to other algorithms, the optimized MLP model exhibited better accuracy and robustness, with a prediction accuracy of up to 95% for the PSO-MLP model, which can meet practical applications.

### 3.3. PSO-MLP Model Optimization Results

**Figure 13:**
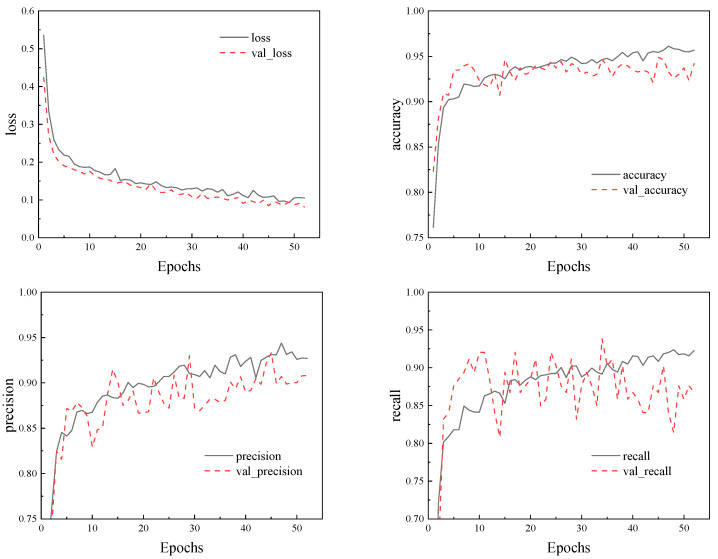
Training metrics for the final MLP model.

After 52 iterations of optimization, the MLP model architecture and parameter values obtained are shown in Table 6. The training process indicators of the final model are shown in Figure 13, without overfitting phenomenon, and the accuracy and precision of the model on both the training and validation sets are high.

### 3.4. Feature Analysis

**Figure 14:**
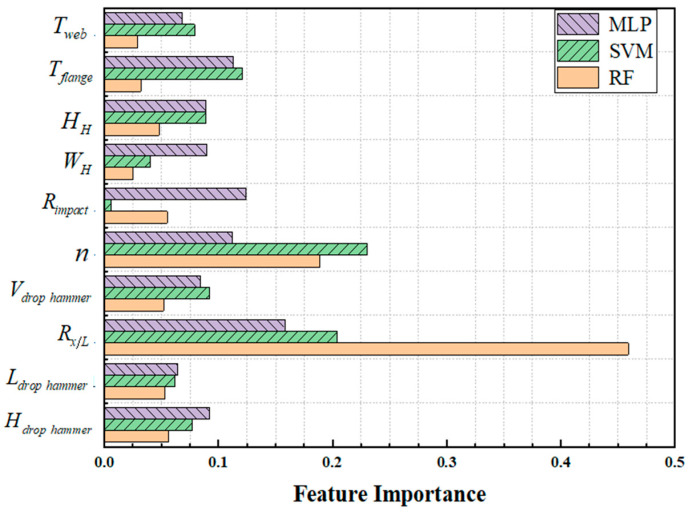
Feature importance factor.

To explain the prediction process of the model, the RF, SVM, and PSO-MLP models were selected for feature importance output and normalized, as shown in Figure 14. In the RF model prediction process, the importance of features *R_x/L_* and *n* exceeds 65%, while the influence of other features is relatively small, indicating that the RF model believes that the values of these two features can largely determine whether the member fails. In SVM and MLP models, the importance of each feature is relatively balanced, which means that when the model predicts whether the member will fail, it will comprehensively consider the values of each feature.

In the MLP model, the importance coefficients of *R_x/L_* and *R_impact_* in the external load characteristics are relatively high, indicating that there are certain differences in the impact resistance performance of the H-shaped steel members at different positions. That is, under the same other impact conditions, the impact characteristic positions are more likely to cause failure. The impact angle indicates that there are significant differences in the impact resistance performance of H-shaped steel members in different directions. The importance of the feature *T_flange_* in the cross-sectional features is relatively high, indicating that the flange thickness of H-shaped steel members has a greater impact on the failure of the members under impact compared to other cross-sectional parameters.

**Figure 15:**
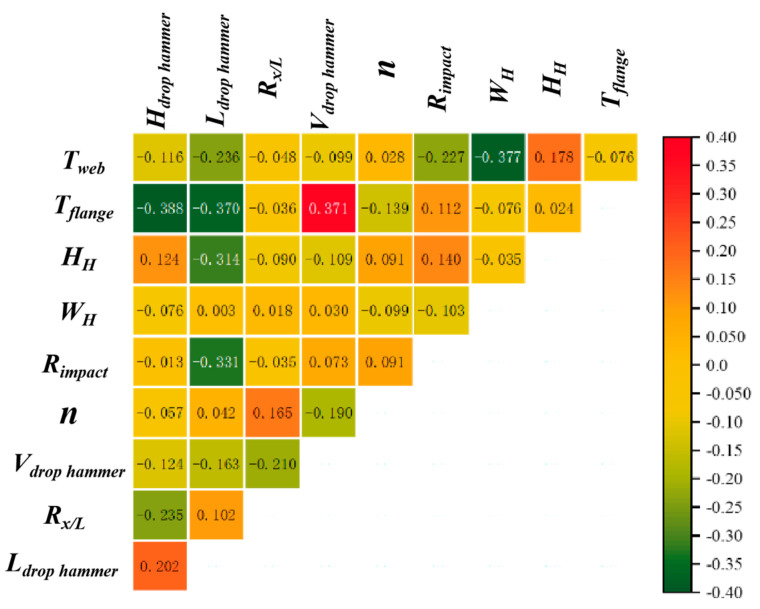
Feature correlation coefficient of MLP.

Figure 15 shows the feature correlation coefficient matrix of the MLP model, with visible feature correlation coefficients ranging from −0.4 to 0.4. This indicates that there is a weak linear relationship between features, and the independence between features is relatively high, which is beneficial for the stability and interpretability of the model. Additionally, the weak correlation also indicates that the selection of features is relatively reasonable, and there is no problem of information redundancy in features.

In the feature correlation coefficient of the MLP model, the correlation coefficient of (*T_flange_*, *V_drop hammer_*) reaches 0.371, which may indicate a moderate positive linear correlation between the flange thickness and the hammer impact velocity. When the model predicts whether the member will fail, the requirement for hammer impact velocity also increases with the increase in flange thickness. The correlation coefficients of (*T_flange_*, *H_drop hammer_*), (*T_flange_*, *L_drop hammer_*), and (*T_web_*, *W_H_*) are −0.388, −0.370, and −0.377, respectively, indicating a moderate negative linear correlation between these feature pairs. The correlation coefficients between *W_H_* and *n* and the external load characteristics are all at a low level, which may indicate that *W_H_* and *n* have a nonlinear relationship with other features or have a small impact, and the specific impact needs further discussion.

## 4. Discussion

In previous studies, experimental research and batch numerical simulation calculations have been conducted, and machine learning models with good prediction accuracy and robustness have been trained and obtained. To further explain the prediction mechanism of the PSO-MLP model, the effects of external load characteristics and section characteristics on the impact resistance of components were analyzed and discussed by taking the failure and non-failure of members determined by the critical drop hammer impact velocity as the index. After determining the size and density of the hammer, the impact velocity of the hammer at the critical failure state of the member is used to measure the impact resistance of the member. Under the premise of fixed other feature values, a bivariate sample dataset is created by selecting a feature and a hammer impact velocity for continuous values and matching them to each other. The MLP model is used for member failure prediction, and a heatmap of the member feature hammer velocity failure state is drawn. The critical failure boundary of the member in the heat map reflects the predicted path of the model and the influence of features on whether the member fails.

For the convenience of discussion, a basic working condition is given here: the cross-sectional size of H-shaped steel is 200 × 400 × 14 × 12, *H_drop hammer_* = 260 mm, *L_drop hammer_* = 80 mm, *m_drop hammer_* = 312 kg, *R_x/L_* = 0.45, *n* = 0.67, *R_impact_* = 0 *. Subsequent discussions are based on this fundamental working condition.

### 4.1. The Influence of External Load Features

**Figure 16:**
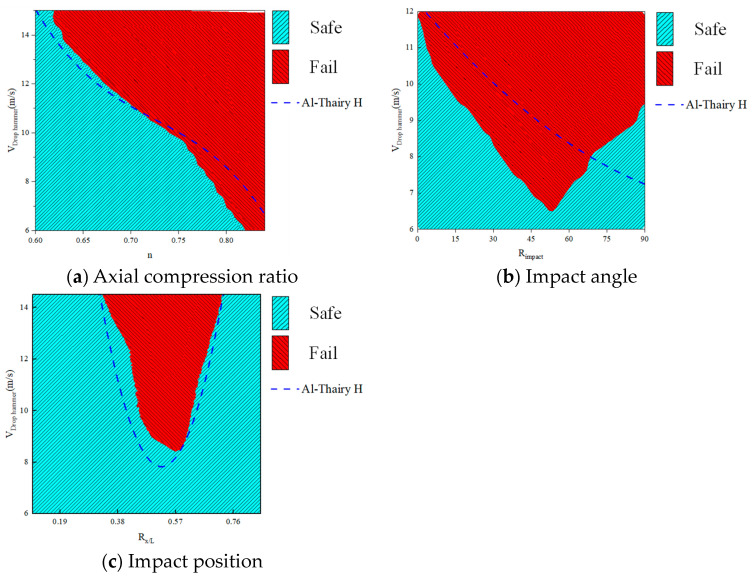
The influence of external load features on the impact resistance of members.

Specific continuous values of *n*, *R_impact_,* and *R_x/L_* were taken and matched with different *V_drop hammer_* values to obtain a large number of samples. The trained PSO-MLP model was used for prediction to obtain the hotspot map of the prediction results, as shown in the figure below. For the convenience of discussion, the serrated boundary has been smoothed. Here, a comparison is made to the critical impact velocity formula for members proposed by Al-Thairy [42]. Under the boundary constraint conditions discussed in this article, the critical impact velocity formula can be summarized as follows:

(13) $$\frac{1}{2}mv_{c}^{2}=\frac{1}{P}\Omega^{2}f_{y}^{2}W^{2}\gamma^{2}{\left(1-n^{\Omega }\right)}^{2}\left[\frac{8}{L}\left(1-\frac{PL^{2}}{2P_{cr}}\right)-\frac{{\left(2L-x^{\prime }\right)}^{2}}{2Lx^{\prime }(L-x^{\prime })}\right]$$

where *P* is the axial pressure exerted on the member; *n* is the axial compression ratio; *x*’ is the distance from the impacted position to the fixed end; *L* is the length of the member; *f_y_* is the yield stress of the steel; Ω and *γ* is the cross-sectional coefficient of the member; *W* is the flexural modulus in the direction of member impact; *P_cr_* is the critical axial force value for Euler buckling. The predicted results of the machine learning model and the calculated results of Formula (13) are shown in Figure 16.

From Figure 16a, it can be seen that as *n* increases, the critical failure of member *V_drop hammer_* roughly decreases linearly, while as *R_impact_* and *R_x/L_* increase, the critical failure of member *V_drop hammer_* shows a phenomenon of first decreasing and then increasing. The influence of axial compression ratio and impact position on the results is consistent with Al-Thairy [42]. However, in the study of the impact angle on the results, existing conclusions indicate that the strong axial strength of the member is stronger than the weak axial strength of the member in terms of impact resistance, while there is relatively little research on other angles. The following Figure 16b indicates that under specific impact conditions, there is a special weak angle, and its impact resistance performance is weaker than that of strong and weak axes. Meanwhile, this also proves that different local deformations caused by impacts can affect the overall performance of members [13,43]. It can be seen from Figure 16c that as the impact position is 0.57 L (*R_x/L_* = 0.57) away from the fixed end, the critical speed is the smallest, so this position is the weakest position.

### 4.2. The Influence of Cross-Sectional Features

**Figure 17:**
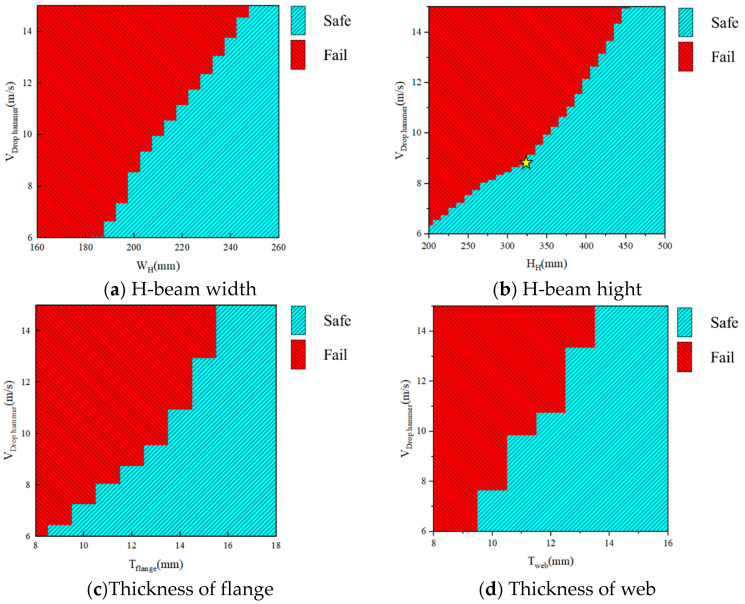
The influence of cross-sectional features on the impact resistance of members.

The same work was carried out on *W_H_*, *H_H_*, *T_flange_*_,_ and *T_web_* shown in Figure 17. It can be seen that as the cross-sectional size increases, the impact resistance performance of the member significantly increases. Among them, the increase in flange thickness and web thickness leads to an increase in the critical failure *V_drop hammer_* of the member, which is similar to previous research results [44]. However, there is relatively little research on the effects of member width and height. The *V_drop hammer_* is linear enhanced with the section width, as the Figure 17a. But for the section height, the linear relation is been divided into two phrases, as the sign point in Figure 17b, which the rate is higher when the section height is larger than 325mm. The reason is the property such as bending resistance is not totally linear related to section height. If the web height-to-thickness ratio is too large, local web buckling could reduce the structural integrity, particularly under axial loads. This limitation should be carefully considered in practical engineering applications.

## 5. Conclusions

The effectiveness and robustness of combining experiments, machine learning, with batch numerical simulation to predict the failure state of H-shaped steel components are investigated in this paper. The key innovation of this study lies in the construction of a prediction model based on MLP, which can qualitatively predict whether different H-shaped steel members would fail under different impact conditions. The MLP model in this article uses the PSO meta-heuristic algorithm to optimize hyperparameters for better accuracy, precision, and recall. Moreover, the MLP model was compared and analyzed to PR, RF, SVM, and KNN models. The main conclusions drawn from this article are as follows:

(1) Due to the complexity of factors affecting the impact resistance of H-shaped steel members, traditional research methods make it difficult to consider numerous variables, especially in qualitatively judging the state of members under specific impact conditions;
(2) The PSO-MLP model proposed in this article provides a convenient solution for qualitatively judging the results and evaluation of H-shaped steel members under specific impact conditions. Unlike the traditional methods, it avoids complex formulas or the selection of specific parameters and only needs to predict the impact results through the model based on the member size and possible impact load parameters. It exhibits excellent prediction accuracy and robustness in the prediction set, with higher accuracy, precision, and recall compared to PR, RF, SVM, and KNN models;
(3) Based on the MLP model trained in this paper, the prediction of sample states under specific impact conditions was carried out. The impact performance was evaluated using the drop hammer velocity at the critical failure of the member, and the impact of different feature changes on the impact performance was analyzed. The weakest impact location was 0.57 L away from the fixed end, and the effects of axial compression ratio, flange, and web thickness were similar to previous research results. There was a strong nonlinear relationship between the impact angle and the critical impact velocity. The height and width of the cross-section showed a linear improvement in impact resistance performance.

While we have reached certain findings regarding the impact failure of H-section steel members, these findings necessitate validation through additional research. The model used in the study can be further refined and expanded, taking into account more influencing factors such as complex boundary conditions and different hammer shapes to enhance the versatility and accuracy of the model. Exploring new machine learning algorithms, like those based on physical information fusion, can improve the predictive performance and generalization ability of the model. At the same time, developing interpretive tools for the model will increase its transparency and credibility.

## Figures and Tables

**Table 1:** Measured performance parameters of steel.

Nominal Thickness (mm)	Yield Stress (MPa)	Young’s Modulus (GPa)	Ultimate Stress (MPa)
4	403.13	200.41	553.75
5	383.38	188.21	529.19
6	379.65	185.21	522.42
8	369.54	177.05	504.22

**Table 2:** Test member parameters.

Specimens	h × b × t_w_ × t_f_	L (mm)	*n*	V (m/s)	E_0_ (kJ)
1	225 × 150 × 4 × 8	1500	0.2	10	12.5
2	225 × 150 × 5 × 8	1500	0.2	10	12.5
3	225 × 150 × 5 × 8	1500	0.2	8	8
4	225 × 150 × 6 × 8	1500	0.2	10	12.5
5	225 × 150 × 6 × 8	1500	0.3	10	12.5
6	200 × 150 × 4 × 5	1500	0.2	10	12.5
7	200 × 150 × 4 × 6	1500	0.2	10	12.5
8	200 × 150 × 4 × 6	1500	0.2	8	8
9	200 × 150 × 4 × 8	1500	0.2	10	12.5
10	200 × 150 × 4 × 8	1500	0.3	10	12.5
11	200 × 200 × 6 × 6	1500	0.2	10	12.5

**Table 3:** Member characteristics and value range.

External Load Features	Value Range	Cross-Sectional Features	Value Range
$H_{drop\;hammer}$	[100 mm, 300 mm, 10 mm]	$W_{H}$	[150 mm, 270 mm, 5 mm]
$L_{drop\;hammer}$	[80 mm, 240 mm, 10 mm]	$H_{H}$	[200 mm, 500 mm, 20 mm]
$R_{x/L}$	[0.1, 0.9, 0.02]	$T_{flange}$	[8 mm, 20 mm, 1 mm]
$V_{drop\;hammer}$	[6 m/s, 15 m/s, 0.1 m/s]	$T_{web}$	[8 mm, 20 mm, 1 mm]
$R_{impact}$	[0°, 90°, 1°]		
n	[0.1, 0.9, 0.05]		

**Table 4:** Comparison of experimental and numerical simulation results.

Specimens	1	2	3	4	5	6	7	8	9	10	11
$\delta_{L-\mathrm{Test}}$/mm	39.8	26.1	14.7	15.6	16.1	53.1	47.9	28.5	32.9	49.9	14.5
$\delta_{L-\mathrm{Lsdyna}}$/mm	36.8	27.6	15.5	16.2	14.8	50.7	45.0	30.2	30.2	53.9	15.9
Error rate/%	−7.5	5.7	5.4	3.8	−8.1	−4.5	−6.1	6.0	−8.2	8.0	6.7
$\delta_{G-\mathrm{Test}}$/mm	14.7	13.4	8.5	12.9	14	40.5	23.8	15	21.3	30.5	14.8
$\delta_{G-\mathrm{Lsdyna}}$/mm	15.5	12.9	7.9	11.8	14.1	43.2	24.5	14.7	20.2	33.2	14.0
Error rate/%	5.4	−3.7	−7.1	−8.5	0.7	7.9	2.9	−2.0	−5.2	8.9	−5.4

**Table 5:** Comparison to other algorithmic models.

Model	PSO-MLP	RF	SVM	PR	KNN
Accuracy	0.97	0.85	0.91	0.77	0.87
Precision	0.96	0.76	0.86	0.67	0.81
Recall	0.94	0.72	0.85	0.75	0.68

**Table 6:** MLP model hyperparameter results.

Model Parameter	Hyperparameter Value
Number of input layer neurons	10
Number of hidden layers	3
Number of hidden layer neurons	(20, 20, 26)
Hidden layer activation function	Sigmoid
Dropout_rate	0.169
Batch_size	72

## Data Availability

The original contributions presented in this study are included in the article. Further inquiries can be directed to the corresponding author.
